# Removal of methylene blue dye by solvothermally reduced graphene oxide: a metal-free adsorption and photodegradation method†

**DOI:** 10.1039/c9ra05793e

**Published:** 2019-11-19

**Authors:** Valerie Ling Er Siong, Kian Mun Lee, Joon Ching Juan, Chin Wei Lai, Xin Hong Tai, Cheng Seong Khe

**Affiliations:** Nanotechnology & Catalysis Research Centre, Institute for Advanced Studies, University of Malaya Kuala Lumpur Malaysia cwlai@um.edu.my; Department of Fundamental and Applied Sciences, Universiti Teknologi PETRONAS Perak Darul Ridzuan Malaysia

## Abstract

In this work, reduced graphene oxide (rGO) was fabricated at different reduction temperatures *via* an environmentally friendly solvothermal approach. The rGO formed at 160 °C clearly showed the partial restoration of the sp^2^ hybridization brought about by the elimination of oxygenated functionalities from the surface. Owing to the augmented surface area and the band gap reduction, rGO-160 exhibited the best adsorption (29.26%) and photocatalytic activity (32.68%) towards the removal of MB dye. The effects of catalyst loading, initial concentration of dye, light intensity, and initial pH of solution were evaluated. It was demonstrated that rGO-160 could achieve a higher adsorptive removal (87.39%) and photocatalytic degradation (98.57%) of MB dye when 60 mg of catalyst, 50 ppm of dye at pH 11, and 60 W m^−2^ of UV-C light source were used. The MB photodegradation activity of rGO-160 displayed no obvious decrease after five successive cycles. This study provides a potential metal-free adsorbent-cum-photocatalyst for the decontamination of dyes from wastewater.

## Abstract 2

A metal-free MB dye removal process was carried out by solvothermally synthesized rGO. After optimization, near-complete dye removal was achieved *via* an adsorption and UV photodegradation route.

## Graphical abstract

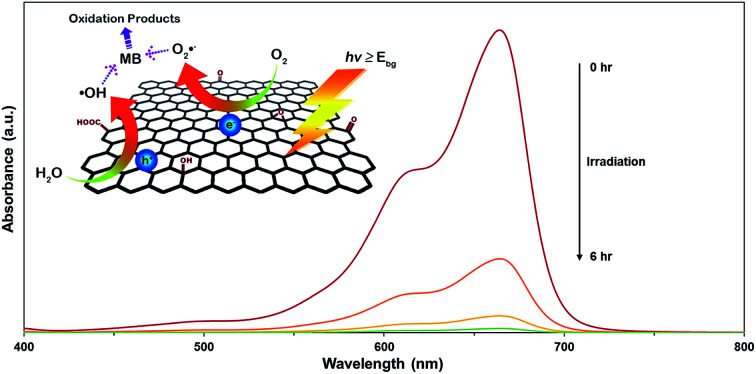

## 1. Introduction

Dyes play an important part in different areas of the textile manufacturing industries. Nevertheless, the dyes are non-biodegradable, and are often released into the water environment without proper precautions.^1^ Most of the dyes possess toxic and carcinogenic properties, which can have deleterious effects on human health and ecosystems.^2^ Dyes are stable towards natural degradation processes due to their intricate aromatic structures.^3^ For this reason, the removal of dyes has been accomplished by various treatment techniques. The adsorption process is by far the most common approach used in industry, due to the accessibility of inexpensive adsorbents with high adsorption capacities.^4^ The photocatalysis process is yet another popular method used due to its easiness of application.^5^ Generally, both superoxide (·O_2_^−^) and hydroxyl (·OH) radicals are widely accepted as the reactive oxygen species (ROS) in the photooxidation of dye pollutants.^6^ The elimination of dyes in both reactions is highly dependent on the surface area of the materials used.^7^

Being a type of chemically-altered graphene, reduced graphene oxide (rGO) is more economically fitted for extensive production than pristine graphene.^8^ Commonly, rGO finds its application in the manufacture of graphene-based composites.^9^ rGO can be fabricated in various ways, including microwave, thermal, photo-thermal, chemical, photo-chemical, as well as microbial/bacterial processes.^10^ Chemical method appears to be advantageous owing to its affordability, simplicity, and extensive production.^11^ Particularly, there are three main steps in preparing rGO *via* this method. The initial step refers to the formation of graphite oxide from graphite by oxidation, whereby the surficial oxygen functionalities are introduced to the graphene layers. Due to the presence of oxygenated functionalities like carboxyls, hydroxyls and epoxides on the surface, graphite oxide is able to disperse in polar solvents, thereby forming stable dispersions. Subsequently, graphite oxide is exfoliated by either sonication or mechanical stirring to create graphene oxide (GO) with single or few layers. Lastly, GO is reduced to rGO by eliminating the surficial oxygen functionalities.^12^

Carbon-based materials have been traditionally utilized in the adsorption reactions to eliminate the organic and inorganic contaminants.^13,14^ One of the most popular adsorbents is the activated carbon.^15^ Recently, rGO has shown increasing usage in dye adsorption application.^16,17^ Owing to the presence of remaining surficial oxygen functionalities, along with some defects in the graphitic domains, rGO is deemed efficient for the adsorptive removal of dyes.^18^ Generally, rGO interacts with dyes through π–π interaction, electrostatic interaction, hydrophobic association and structural conjugation. These interactions enable a wide range of dyes to adsorb on rGO.^19^ In order to improve the dye adsorption capacity, rGO with large surface area, together with high porosity, is preferable, and this is achievable by controlling the quality of the GO precursor and the reduction method used.^20^

Today, the photocatalytic degradation of dyes has been used as a tool for manifesting the technological benefits of photocatalysis. Generally, when the band gap of a photocatalyst is equal to or lower than the energy of light absorbed, electrons are excited, thereby forming electrons and holes. The surrounding oxygen and water molecules are then utilized by the photogenerated electron–hole pairs to produce ROS, which are responsible to break down the dye molecules.^21^ There have been various studies reporting the usage of rGO in the elimination of dye pollutants by means of photocatalysis, especially the removal of harmful methylene blue (MB) dye from wastewater (Table S1†). In spite of the fact that rGO exhibited outstanding MB adsorption ability, there is a lack of information about the optimization of its photoactivity. Since rGO behaves like a semiconductor, plus the fact that it is inexpensive and friendly to the environment, there is a great potential for utilizing rGO as a metal-free photocatalyst for environmental remediation.

In this work, rGO with excellent adsorption properties and photoactivity towards the removal of MB dye was synthesized *via* an eco-friendly and simple solvothermal technique without employing any toxic reducing agents. The optimization of extraneous factors such as amount of catalyst, initial dye concentration, intensity of light, and pH were investigated.

## 2. Experiment

### 2.1. Materials

Synthetic graphite powder with the size of <20 μm was acquired from Sigma Aldrich. Methylene blue (MB, C.I. 52015, ≥ 99%), hydrochloric acid (HCl, ≥ 37%), sulphuric acid (H_2_SO_4_, ≥ 95–97%), hydrogen peroxide (H_2_O_2_, ≥ 30%), and potassium permanganate (KMnO_4_, ≥ 99%) were obtained from Friendemann Schmidt. Di-phosphorus pentoxide (P_2_O_5_, ≥ 99%), potassium peroxodisulphate (K_2_S_2_O_8_, ≥ 99%), and ethanol (C_2_H_5_OH, ≥ 99.9%) were acquired from Merck. The materials used were all of analytical grade. In all experiments, deionized water (18.2 MΩ cm) was employed.

### 2.2. Pre-treatment of graphite powder

Initially, graphite (3 g) was added into H_2_SO_4_ (12 ml) containing P_2_O_5_ (2.5 g) and K_2_S_2_O_8_ (2.5 g).^22^ The mixture was stirred for a period of 4.5 hours at 80 °C in an oil bath. Then, the mixture was gradually cooled down to ambient temperature. The mixture was kept aside for one night with the addition of deionized water (500 ml). After vacuum filtration, the residue was washed with deionized water prior to drying.

### 2.3. Preparation of graphene oxide (GO)

A modified Hummers' method was used to oxidize graphite to GO.^23^ In a typical process, pre-treated graphite was mixed with H_2_SO_4_ (120 ml) in an ice bath and vigorously stirred. The mixture was gradually added with KMnO_4_ (15 g) and kept at 35 °C for 2 hours. The stirring was continued for another 2 hours after deionized water (250 ml) was added to the mixture. Then, deionized water (500 ml) was added again, before adding 30% H_2_O_2_ (20 ml) solution to cease the reaction. After the mixture was centrifuged, the GO precipitate obtained was cleansed with 10% (v/v) HCl solution, followed by deionized water *via* centrifugation until a final pH of about 4 was obtained. Finally, GO was dried, pestled, and stored.

### 2.4. Preparation of reduced graphene oxide (rGO)

An eco-friendly and facile solvothermal approach was used to reduce GO to rGO. Initially, GO (200 mg) was mixed with deionized water (10 ml) and C_2_H_5_OH (20 ml). A stable GO dispersion was obtained after 30 minute of rigorous sonication of the mixture. Then, the mixture was transferred into an 80 ml Teflon-lined stainless-steel autoclave, and heated at different temperatures (80, 120, 160 and 180 °C) for 2 hours. The samples were labelled accordingly based on the denotation of rGO-*x*, where *x* represents the studied reduction temperatures. Subsequently, the as-obtained rGO samples were filtered, rinsed, and dried.

### 2.5. Methods of characterization

The ordered and disordered crystalline structures of GO and rGOs were determined *via* Raman spectroscopy by employing Renishaw inVia Raman Microscope (Gloucestershire, UK) equipped with Ar-ion laser (*λ* = 514 nm) over a scan range of 100–3200 cm^−1^. The elemental atomic composition of GO and rGOs were analyzed *via* energy dispersive X-ray spectroscopy (EDX) by using FEI Quanta FEG 650 EDX Unit (Oregon, USA). The crystalline phases of GO and rGOs were investigated *via* X-ray diffraction (XRD) by using PANalytical X-ray Diffractometer (Almelo, Netherlands) with Cu Kα (*λ* = 0.15406 nm) radiation over a scan rate of 0.02° s^−1^ at a 2*θ* range of 10–70° under 40 kV/30 mA. Brunauer–Emmett–Teller (BET) and Barrett–Joyner–Halenda (BJH) analyses were carried out in N_2_ atmosphere at 77 K using Micromeritics ASAP 2020 Surface Area and Porosity Analyzer (Georgia, USA) to examine the specific surface areas, pore sizes, and pore volumes of GO and rGOs. Degassing of the samples was performed at 150 °C for 24 hours prior to measurement to remove moisture. Ultraviolet-visible (UV-vis) spectrophotometry was conducted by using PerkinElmer Lambda 35 UV/vis Spectrophotometer (Massachusetts, USA) at an absorption range of 200–800 nm to elucidate the optical properties of GO and rGOs. The optical band gaps of GO and rGOs were then determined from the linear extrapolation of Tauc plots.

### 2.6. Adsorption and photocatalytic activity measurements

**Fig. 1:**
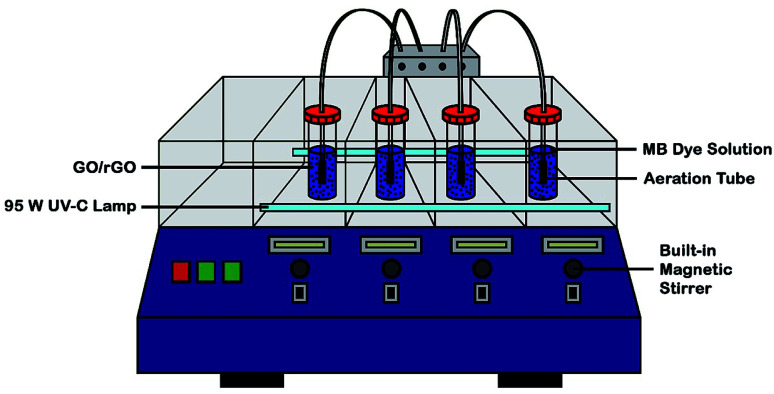
Schematic diagram of custom-made UV-C photoreactor.

In a custom-made photoreactor (Fig. 1), GO/rGO (20 mg) suspended in MB solution (50 ppm, 100 ml) was aerated and stirred in darkness to determine the time needed to achieve dye adsorption–desorption equilibrium. Then, a new and similar suspension was allowed to reach dark adsorption and desorption equilibrium, followed by 6 hours of irradiation with UV-C light (2 × 95 W; intensity ≈ 60 W m^−2^). At certain intervals, aliquots were withdrawn, filtered, and subjected to UV-vis analysis at 664 nm. The photocatalyst with the best adsorption and photoactivity was then optimized based on catalyst dosage, initial concentration of dye, light intensity, and pH of solution. In addition, control experiment was also conducted prior to adsorption and photocatalytic activity measurements, whereby MB solution (50 ppm, 100 ml) was irradiated with the same UV-C light in the absence of photocatalyst for 10 hours. Under optimized experimental conditions, the stability and reusability of the optimum rGO sample towards the MB photodegradation process were determined by repeating the reaction for up to five cycles.

The adsorption percentage (% *C*_ads_) and the photodegradation efficiency (% *C*_deg_) were calculated accordingly by using the following equations:1
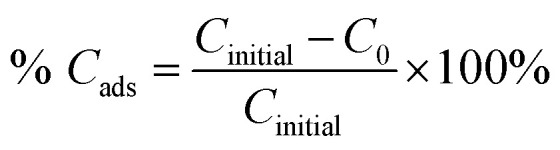
2
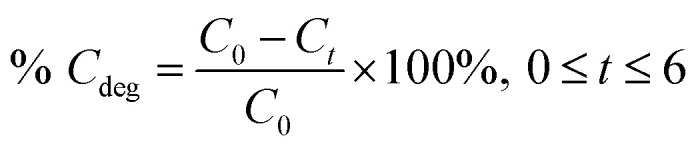
where *C*_initial_, *C*_*0*_, and *C*_*t*_ are the respective concentrations of MB dye at the initial, after reaching adsorption–desorption equilibrium, and at time, *t*. Subsequently, the photocatalytic degradation reaction was modeled by employing the pseudo-first-order kinetic equation as below:3
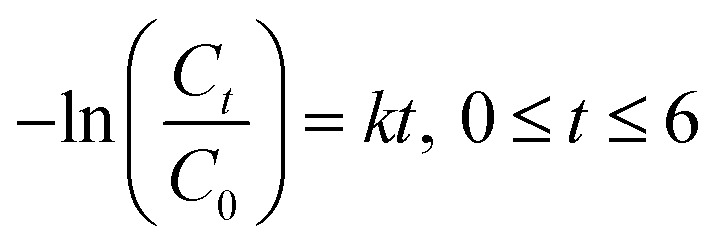
where *k* (h^−1^) denotes the rate constant for pseudo-first-order.

## 3. Results and discussion

### 3.1. Characterization of GO and rGOs

**Fig. 2:**
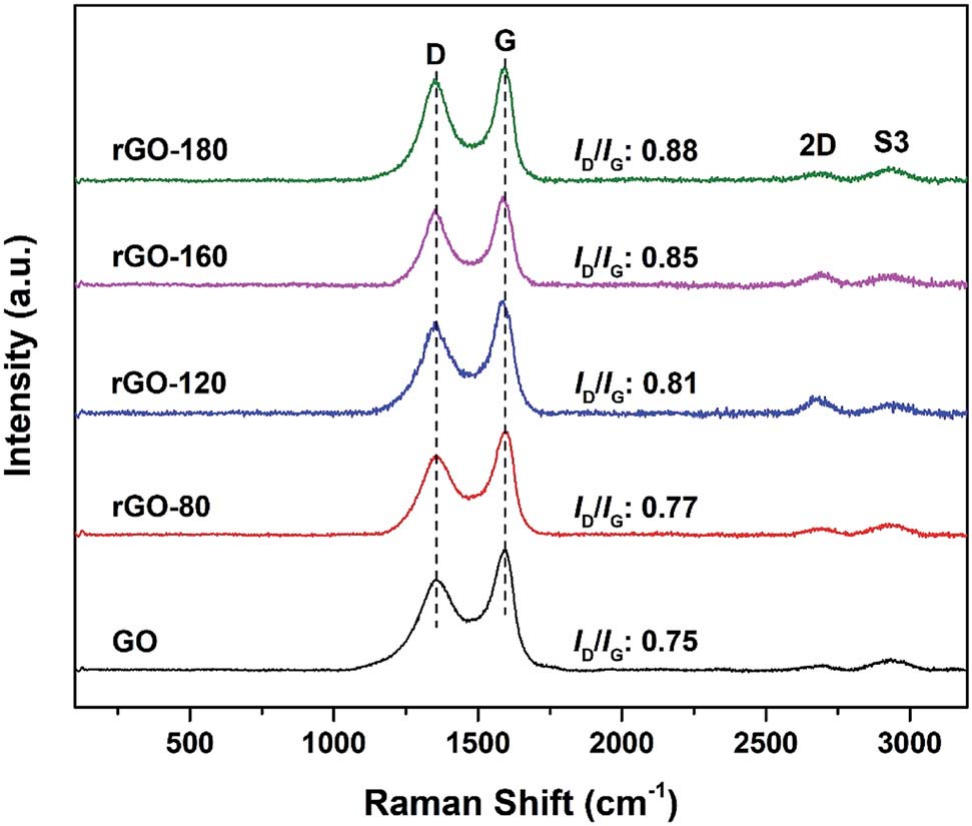
Raman spectra of GO, rGO-80, rGO-120, rGO-160 and rGO-180.

The Raman spectra of GO and rGOs are shown in Fig. 2. All samples exhibited both D (∼1353 cm^−1^) and G (∼1591 cm^−1^) bands of graphene. The D band referred to the breathing mode of sp^2^ carbon atoms in the hexagonal structure.^24^ It was resulted from the formation of structural defects.^25^ The G band reflected the vibration of sp^2^ carbon atoms in the aromatic rings.^26^ In contrast to D band, the G band demonstrated higher intensity, suggesting that the samples were not fully exfoliated.^27^

The intensity ratio of the D to G band (*I*_D_/*I*_G_ ratio) can be used to gauge the degree of disorder and the change in average size of the sp^2^ fraction during the reduction of GO to rGO.^28^ In Fig. 2, the *I*_D_/*I*_G_ ratio increased steadily (from 0.75 of GO to 0.88 of rGO-180) with higher reduction temperature, signifying an increased degree of reduction.^29,30^ This can be due to more defects were created as the surficial oxygen functionalities were gradually eliminated during the solvothermal reduction process.^31,32^ The defects originated from surface ripples and edges, as a result of higher degree of defragmentation and smaller average sp^2^ domain size after reduction.^28,33^ On the other hand, the 2D (∼2684 cm^−1^) and S3 (∼2935 cm^−1^) bands were also detected. The 2D band is attributed to the inelastic scattering from two phonons, whereas the S3 band is resulted from the combination of both D and G bands.^34^ As the reduction temperature increased, the intensities of the two bands also slightly increased, indicating an enhanced degree of graphitization.^35^

Table 1 and Fig. S1† show the EDX elemental atomic analysis of GO and rGOs, where only carbon and oxygen were detected. The C/O atomic ratio of GO is the lowest at 1.79, which implied that higher oxygen content was present in GO. The value is comparable to the findings obtained in previous GO studies.^36,37^ After solvothermal reduction of GO, the C/O atomic ratio was found to increase, owing to the reduced oxygen content.^36,37^ At lower reduction temperatures of 80 °C and 120 °C, the increment in C/O atomic ratio was small. However, as the reduction temperature was elevated to 160 °C and 180 °C, the C/O atomic ratio was drastically increased. The EDX results concurred with the Raman data presented, whereby higher reduction temperature led to higher degree of GO reduction.

**Table 1:** EDX elemental analysis of GO, rGO-80, rGO-120, rGO-160 and rGO-180

Sample	Elemental analysis (at%)		
	C	O	C/O
GO	64.22	35.78	1.79
rGO-80	65.02	34.98	1.86
rGO-120	67.16	32.84	2.05
rGO-160	80.24	19.76	4.06
rGO-180	81.03	18.97	4.27

**Fig. 3:**
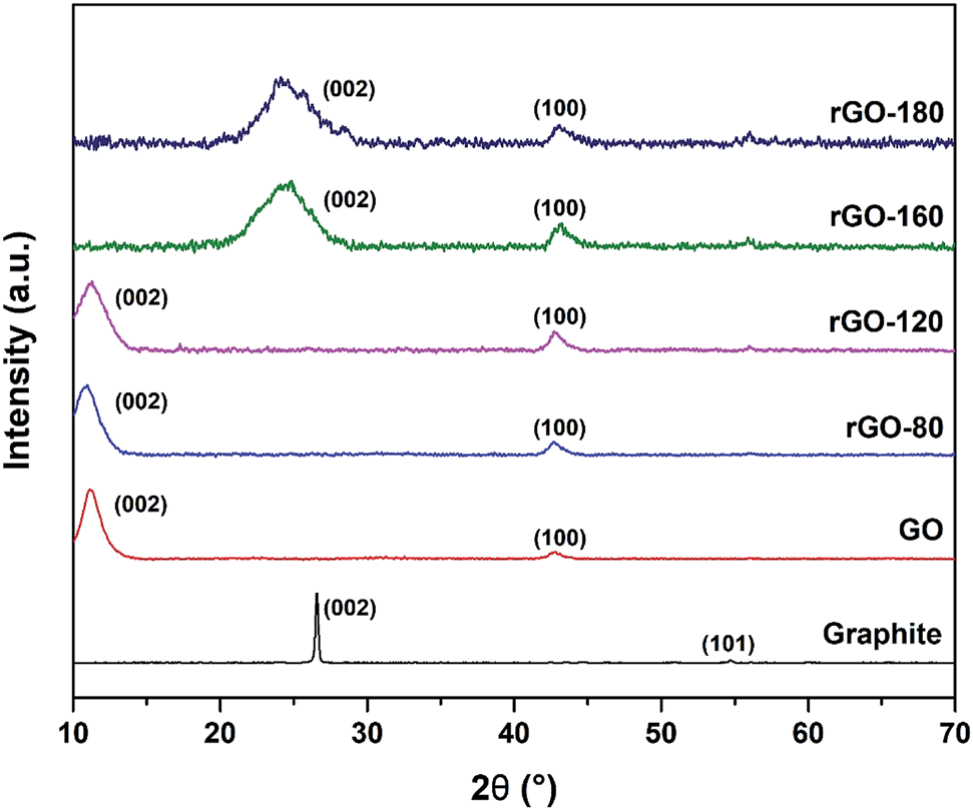
XRD patterns of graphite, GO, rGO-80, rGO-120, rGO-160 and rGO-180.

The XRD diffractograms of graphite, GO and rGOs are depicted in Fig. 3. An intense (002) peak and a very small (101) peak were observed in graphite at 2*θ* = 26.57° and 2*θ* = 54.70°, respectively. Both peaks confirmed the structure of graphite and concurred with JCPDS card of 41-1487.^38^ Upon oxidation, the (002) peak was shifted to 2*θ* = 11.03°, which was attributed to the insertion of oxygenated functionalities and water molecules into the graphene layers.^39^ Nevertheless, the (002) peak of GO was still visible after GO was reduced at 80 °C and 120 °C, possibly due to the low reduction temperatures, which mildly reduced GO to rGO. As the reduction temperature increased, the (002) peak of GO started to disappear while a new broader (002) peak of rGO began to emerge at 2*θ* = 24.89° for rGO-160 and 2*θ* = 23.85° for rGO-180. The shifting of the (002) peak suggested the partial recovery of the sp^2^ carbon structure.^40^ Moreover, the (100) peak of GO at 2*θ* = 42.63° could also be detected in all rGOs, which reflected the turbostratic band of disordered carbon materials.^12^

**Fig. 4:**
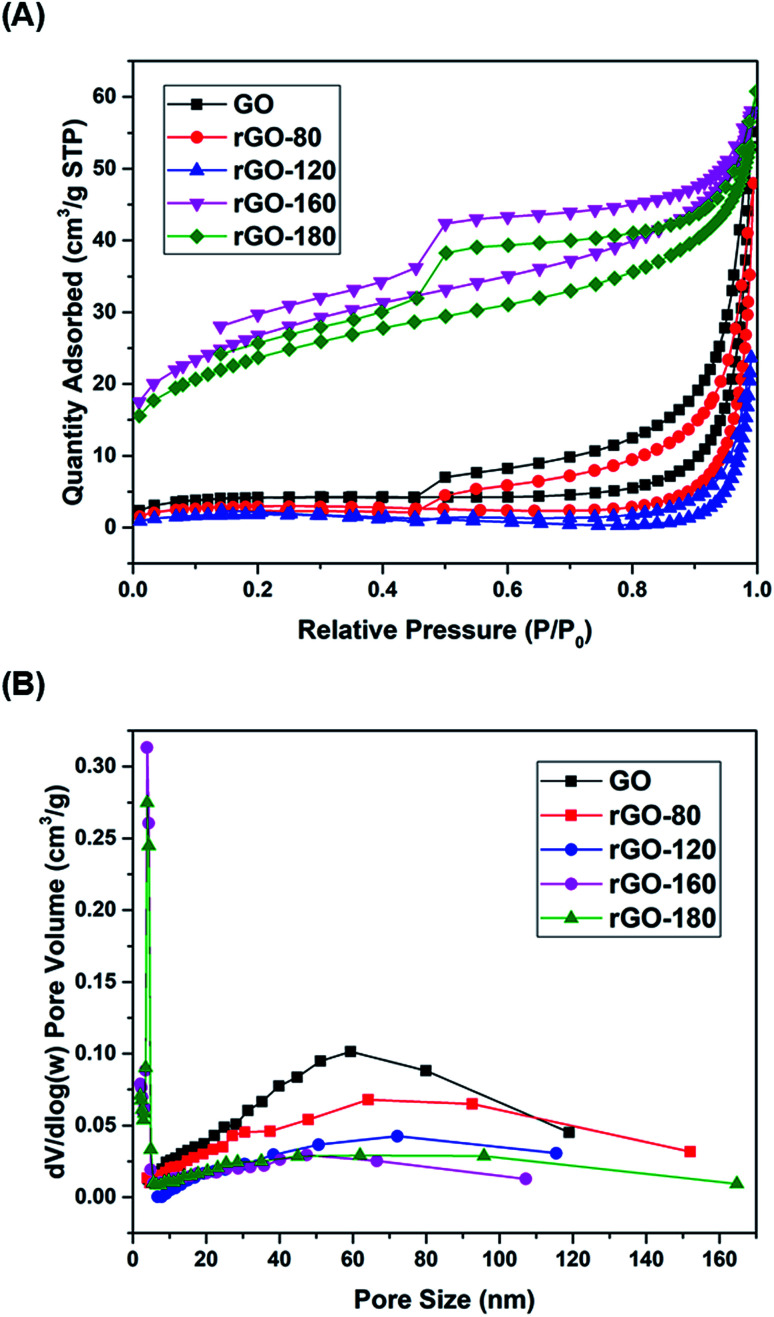
(A) Nitrogen adsorption–desorption isotherms of GO, rGO-80, rGO-120, rGO-160 and rGO-180, (B) pore size distributions of GO, rGO-80, rGO-120, rGO-160 and rGO-180.

The nitrogen physisorption isotherms of GO and rGOs are illustrated in Fig. 4A. All samples were found to show a type H3 hysteresis loop in a type II curve.^41^ A slope was present in each desorption curve, owing to the tensile strength effect that was produced by the instability of meniscus condensation inside the pores.^42^ Both rGO-160 and rGO-180 had hysteresis loops that were partially closed and stopped at around *P*/*P*_0_ = 0.45, possibly due to the presence of micropores in the samples.^43^ The pore size distribution curves of GO and rGOs are demonstrated in Fig. 4B, while the corresponding textural parameters are displayed in Table 2.

**Table 2:** BET/BJH textural parameters of GO, rGO-80, rGO-120, rGO-160 and rGO-180

Sample	Surface area (m^2^ g^−1^)	Pore size (nm)	Pore volume (cm^3^ g^−1^)
GO	15.03	59.42	0.085
rGO-80	10.76	64.19	0.074
rGO-120	6.67	72.18	0.037
rGO-160	94.81	3.89	0.118
rGO-180	83.81	3.90	0.094

Based on Table 2, when GO was reduced from 80 °C to 120 °C, the total pore volume was reduced from 0.085 to 0.037 cm^3^ g^−1^, thus leading to a reduction of surface area. This was probably due to the aggregation effect of GO upon reduction.^44,45^ A pronounced increment of surface area was then observed for rGO-160 at higher reduction temperature, owing to the significant removal of oxygen functional groups,^46^ causing the generation of higher pore volume.^47^ However, when the reduction temperature was increased to 180 °C, the rGO sheets tended to stack together *via* π–π interactions, resulting in the decreased surface area of rGO-180.^48^ Both rGO-160 and rGO-180 exhibited much smaller pore sizes and higher total pore volumes as compared to other samples.

**Fig. 5:**
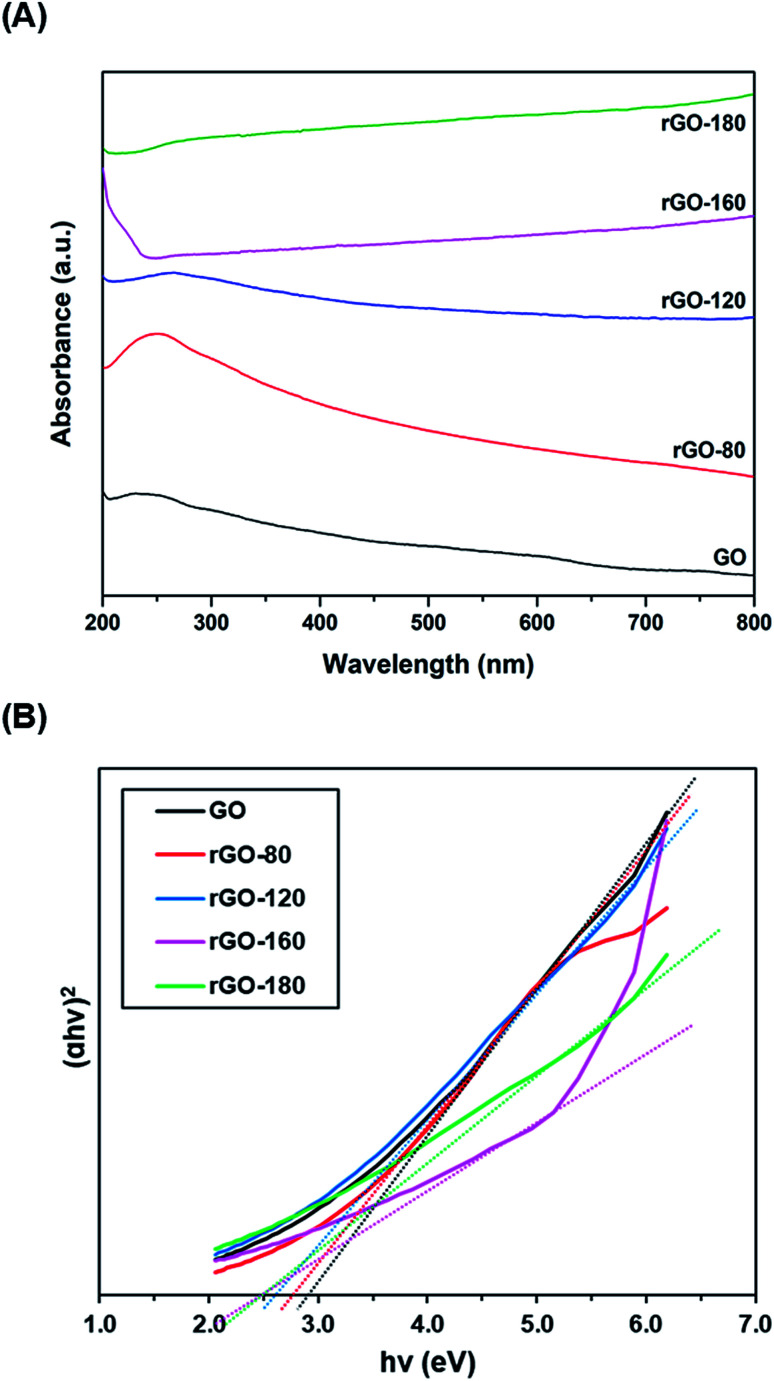
(A) UV-vis absorption spectra of GO, rGO-80, rGO-120, rGO-160 and rGO-180, (B) Tauc plots of GO, rGO-80, rGO-120, rGO-160 and rGO-180.

Fig. 5A shows the UV-vis absorption spectra of GO and rGOs. At 230 nm, a prominent absorption peak was observed in GO, which corresponded to the π–π* transition of aromatic C=C bonds.^49^ When GO was reduced from 80 °C to 180 °C, the peak location slowly moved to higher wavelengths. The red shifting of the peak indicated the partial restoration of the sp^2^ conjugation, thereby reducing the band gap.^50^ The optical band gaps of GO and rGOs were then determined from the linear fits of the Tauc plots, as demonstrated in Fig. 5B. The band gap of GO was about 2.90 eV, concurring with the values reported in the existing literature.^51,52^

### 3.2. Adsorption and photocatalytic activity measurements of GO and rGOs

During the control experiment in the absence of photocatalyst, it was found that only 7.9% of MB dye was removed (Fig. S2†). The slight removal of dye was due to photolysis effect, whereby similar results were also reported in previous studies.^53,54^

**Fig. 6:**
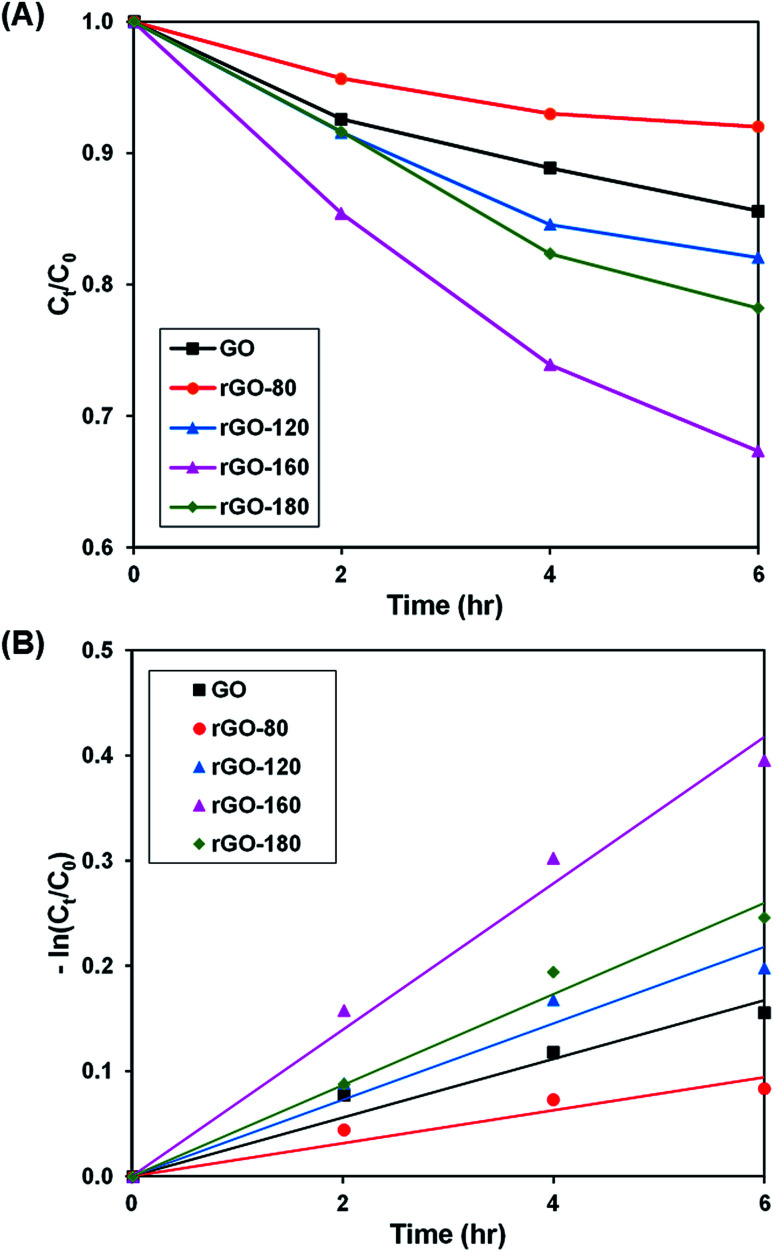
(A) Photocatalytic degradation of MB dye normalized against adsorption, (B) pseudo-first order kinetic plot of MB dye, in the presence of GO, rGO-80, rGO-120, rGO-160 and rGO-180.

Upon stirring in the dark, the dye adsorption–desorption equilibrium on the photocatalysts was reached after 4 hours (Fig. S3–S6†). The photoactivity of GO and rGOs are illustrated in Fig. 6A and S7,† while the pseudo-first-order fitting of photocatalytic dye degradation is depicted in Fig. 6B. The corresponding % *C*_ads_, % *C*_deg_, *k* and *R*^2^ values are shown in Table 3. Since low reduction temperatures were insufficient for GO reduction, both rGO-80 and rGO-120 behaved more or less like GO in the dye adsorption process as compared to other rGOs. With a large surface area, rGO-160 was able to attain the highest adsorption of dye. When the reduction temperature increased to 180 °C, there was a slight reduction in the amount of dye adsorbed owing to surface area reduction. As the irradiated light contained higher energy than the band gaps of GO and rGOs, electrons and holes were able to be generated, thereby providing ROS to break down the dye. Similar to GO, both rGO-80 and rGO-120 also demonstrated lower photoactivity as compared to other rGOs. The highest photoactivity was then exhibited by rGO-160. It was suggested that the dye molecules could interact better with the ROS when they were adsorbed on the surface as compared to moving freely in the aqueous solution.^55^ As rGO-180 exhibited smaller surface area than rGO-160, the available contact area for photocatalytic degradation reaction was also decreased.

**Table 3:** Effect of reaction temperature on adsorption percentage, photocatalytic degradation efficiency, and photocatalytic degradation rate of MB dye

Sample	Adsorption percentage (%)	Photocatalytic degradation efficiency (%)	*k* (h^−1^)	*R* ^2^
GO	10.07	14.41	0.028	0.9517
rGO-80	17.67	8.00	0.016	0.9075
rGO-120	11.60	17.96	0.036	0.9516
rGO-160	29.26	32.68	0.070	0.9844
rGO-180	22.83	21.80	0.043	0.9823

### 3.3. Optimization of adsorption and photocatalytic activity of rGO-160

#### 3.3.1. Effect of catalyst loading

**Fig. 7:**
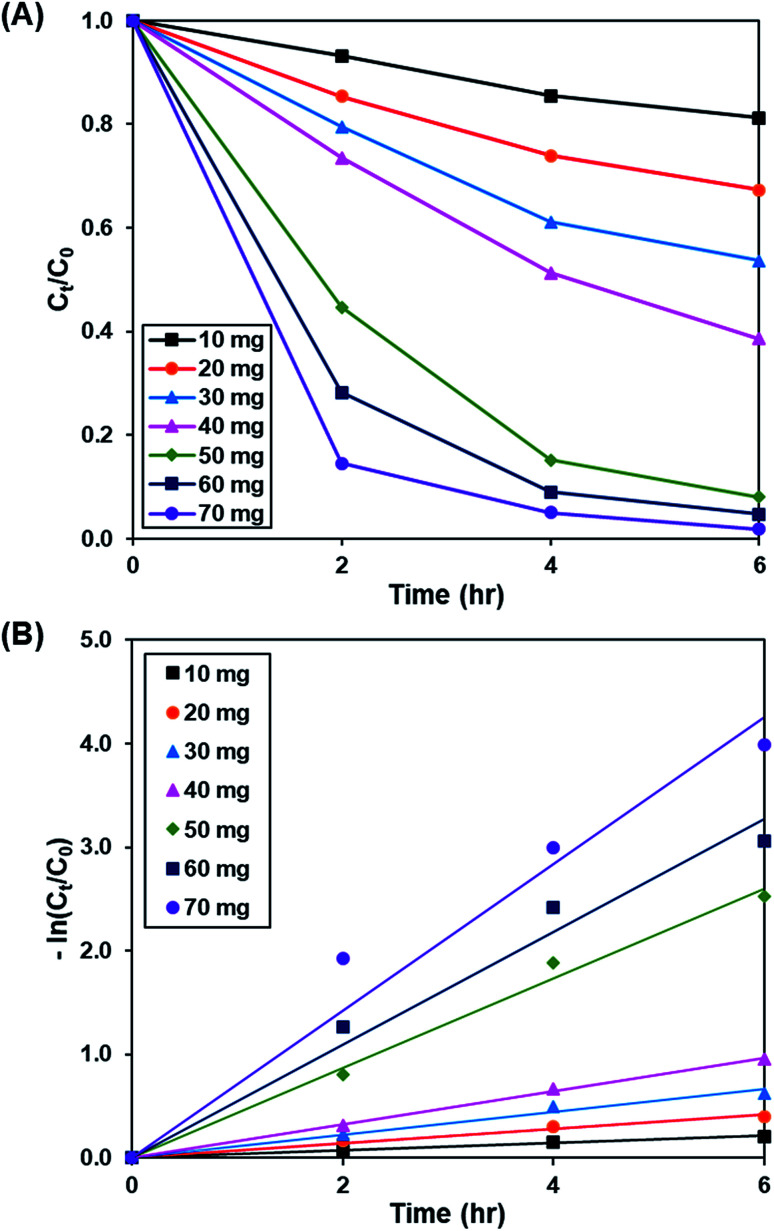
(A) Effect of catalyst loading of rGO-160 on photocatalytic degradation of MB dye normalized against adsorption, (B) pseudo-first order kinetic plot of MB dye, in the presence of 10 mg, 20 mg, 30 mg, 40 mg, 50 mg, 60 mg and 70 mg of rGO-160.

Fig. 7A and S8† demonstrate the effect of catalyst dosage (10 to 70 mg) on the photoactivity of rGO-160, whereas the pseudo-first-order fitting of photocatalytic dye degradation is displayed in Fig. 7B. The corresponding % *C*_ads_, % *C*_deg_, *k* and *R*^2^ values are illustrated in Table 4. The initial concentration of dye (50 ppm), intensity of light (60 W m^−2^), and pH of solution (pH 6) were remained constant at all times. When the catalyst amount was increased, the total surface area of photocatalyst became larger. Subsequently, the available adsorption sites also increased, which in turn improved the dye adsorption process.^56^ Besides that, the total number of catalyst particles also increased, which then enhanced the absorption of photons for the production of electrons and holes, and improved the rate of formation of ROS for the decomposition of dye molecules.^57^ Despite the fact that the highest photoactivity was achieved by using 70 mg of catalyst, the use of such high amount of catalyst almost provoked the complete removal of dye under dark condition. Therefore, 60 mg was considered as the optimum catalyst loading for this work.

**Table 4:** Effect of catalyst loading on adsorption percentage, photocatalytic degradation efficiency, and photocatalytic degradation rate of MB dye

Weight (mg)	Adsorption percentage (%)	Photocatalytic degradation efficiency (%)	*k* (h^−1^)	*R* ^2^
10	10.50	18.82	0.036	0.9919
20	29.26	32.68	0.070	0.9844
30	39.81	46.35	0.110	0.9822
40	50.96	61.46	0.161	0.9983
50	70.72	91.95	0.433	0.9914
60	83.45	95.29	0.545	0.9756
70	91.74	98.15	0.710	0.9594

#### 3.3.2. Effect of initial dye concentration

**Fig. 8:**
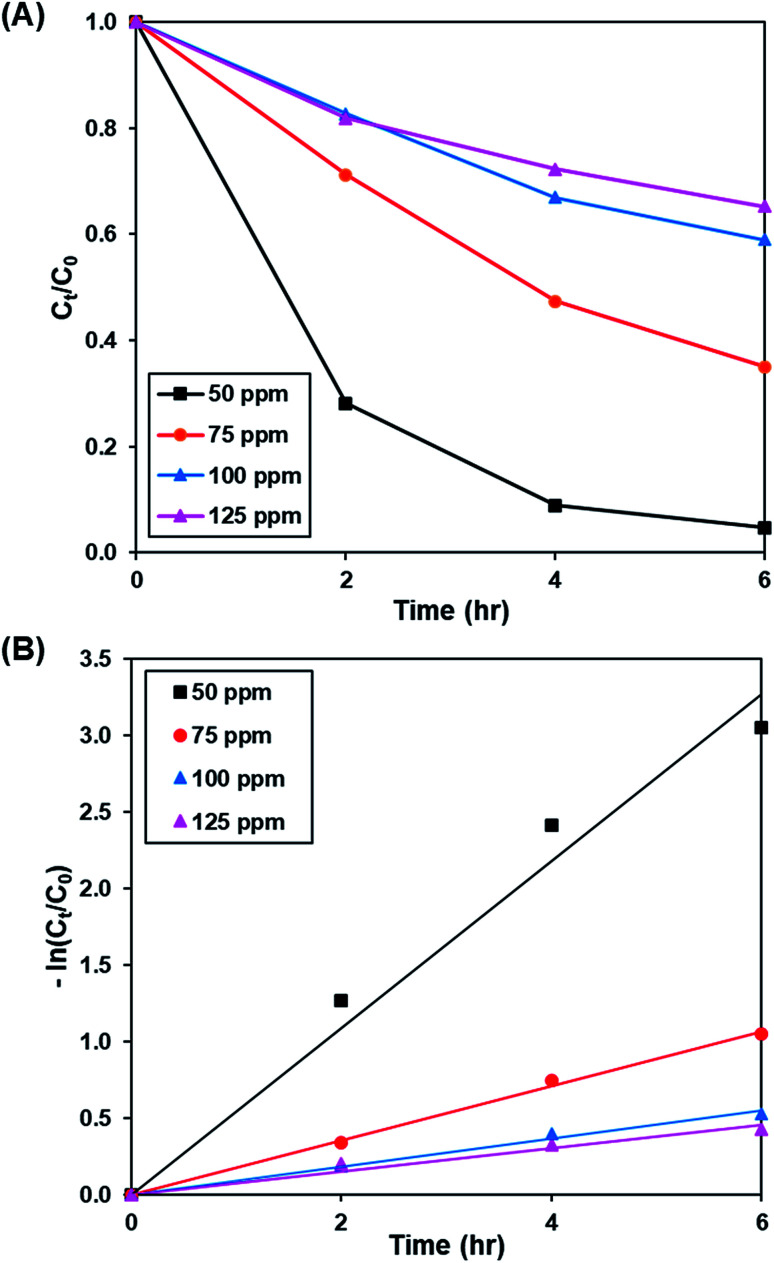
(A) Effect of initial dye concentration of MB solution on photocatalytic degradation of MB dye normalized against adsorption, (B) pseudo-first order kinetic plot of MB dye at 50 ppm, 75 ppm, 100 ppm and 125 ppm of MB solution.

Fig. 8A and S9† show the effect of initial concentration of dye (50 to 125 ppm) on the photoactivity of rGO-160, while the pseudo-first-order fitting of photocatalytic dye degradation is depicted in Fig. 8B. The corresponding % *C*_ads_, % *C*_deg_, *k* and *R*^2^ values are displayed in Table 5. Throughout the experiments, the catalyst loading (60 mg), light intensity (60 W m^−2^), and pH of solution (pH 6) were kept constant. With the increase of initial concentration of dye solution, more dye molecules were accumulated on the catalyst surface. Eventually, the available adsorption sites became saturated and insufficient to accommodate the increasing amount of dye molecules.^58^ The production of ROS for the photocatalytic dye degradation reaction also became limited.^59^ According to the Beer–Lambert law, the photocatalytic degradation rate decreased when the initial concentration of dye increased, owing to the shorter path length of photons entering the solution.^60^

**Table 5:** Effect of initial dye concentration on adsorption percentage, photocatalytic degradation efficiency, and photocatalytic degradation rate of MB dye

Concentration (ppm)	Adsorption percentage (%)	Photocatalytic degradation efficiency (%)	*k* (h^−1^)	*R* ^2^
50	83.45	95.29	0.545	0.9756
75	52.25	64.97	0.178	0.9972
100	40.98	40.99	0.092	0.9893
125	30.14	34.71	0.076	0.9638

#### 3.3.3. Effect of light intensity

**Fig. 9:**
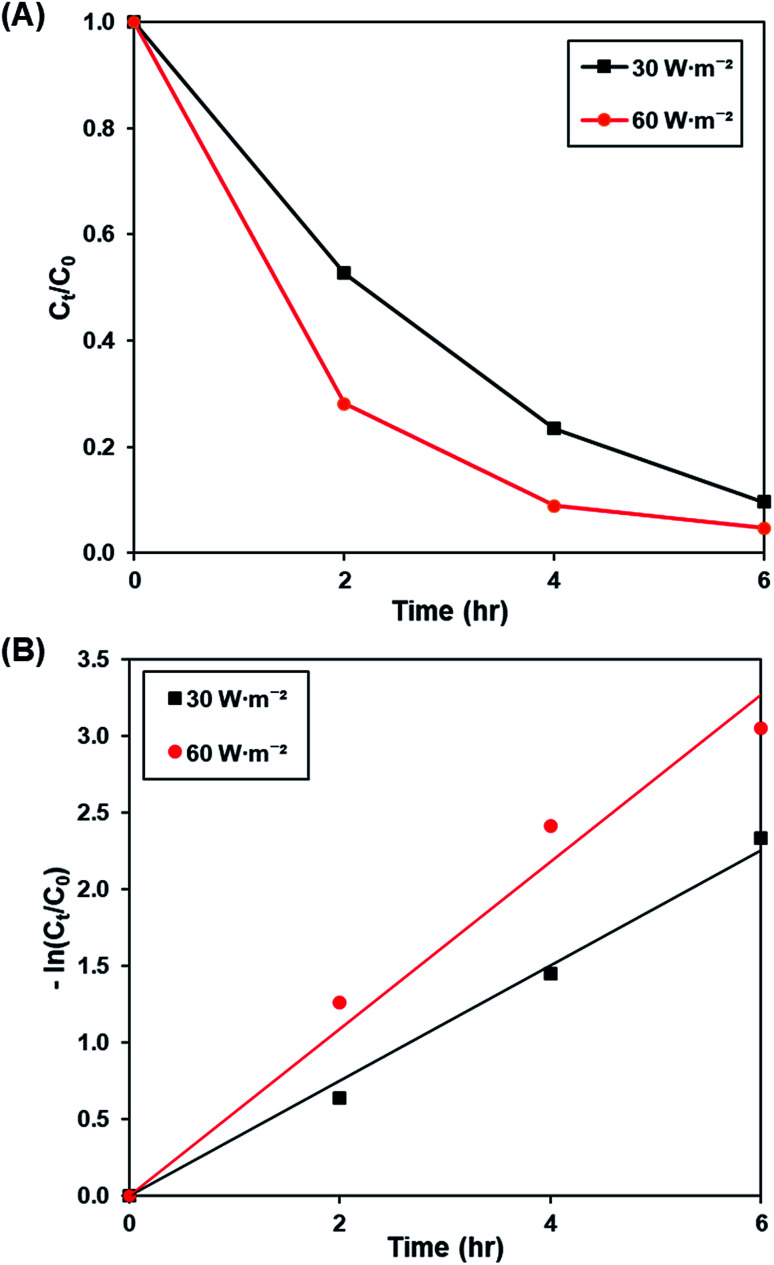
(A) Effect of 30 W m^−2^ and 60 W m^−2^ of UV-C light source on photocatalytic degradation of MB dye normalized against adsorption, (B) pseudo-first order kinetic plot of MB dye at 30 W m^−2^ and 60 W m^−2^ of UV-C light source.

Fig. 9A and S10† illustrates the effect of light intensity (30 W m^−2^ and 60 W m^−2^) on the photoactivity of rGO-160, whereas the pseudo-first-order fitting of photocatalytic dye degradation is shown in Fig. 9B. The corresponding % *C*_ads_, % *C*_deg_, *k* and *R*^2^ values are demonstrated in Table 6. It should be noted that the catalyst loading (60 mg), initial concentration of dye (50 ppm), and pH of solution (pH 6) were kept constant at all times. When the light intensity was increased, there were more photons generated and absorbed by the photocatalyst. As a result, more electrons were readily excited to form more electrons and holes, which created more ROS for the breakdown of dye molecules.^61^

**Table 6:** Effect of light intensity on photocatalytic degradation efficiency and photocatalytic degradation rate of MB dye

Light intensity (W m^−2^)	Photocatalytic degradation efficiency (%)	*k* (h^−1^)	*R* ^2^
30	90.31	0.377	0.9931
60	95.29	0.545	0.9756

#### 3.3.4. Effect of pH

**Fig. 10:**
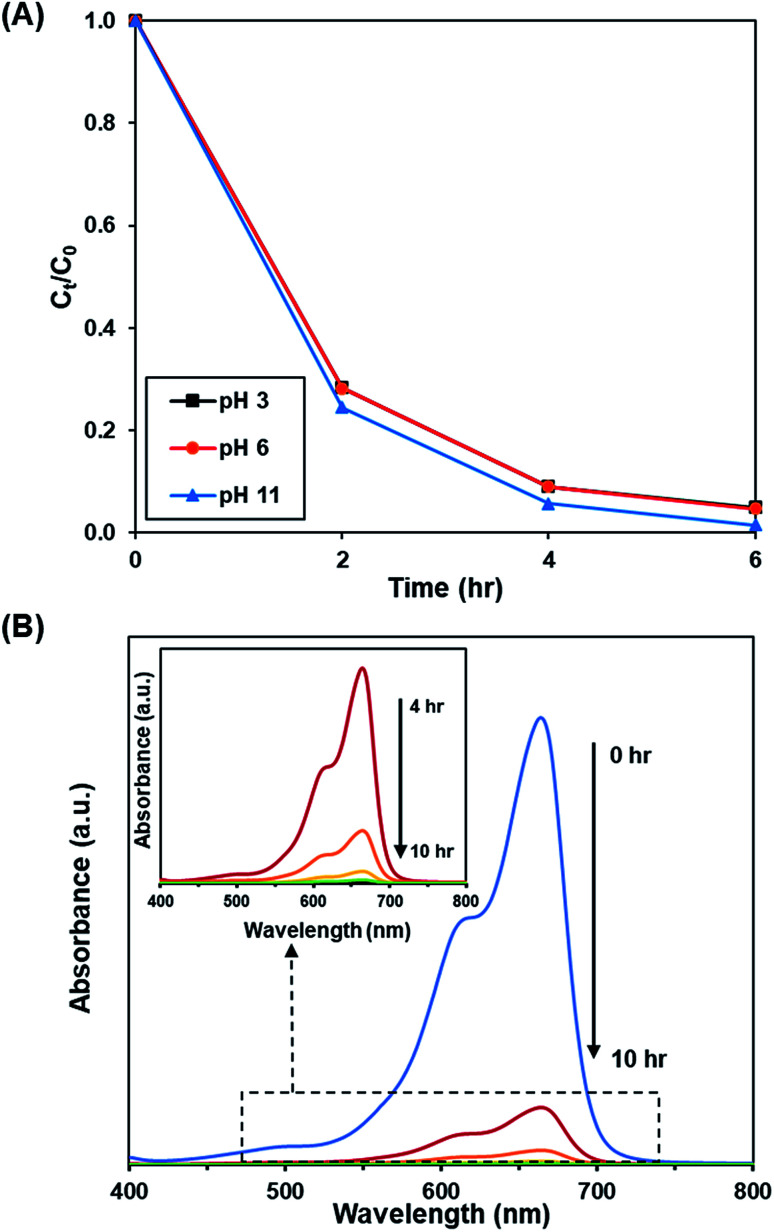
(A) Effect of initial pH of dye solution at 3, 6, and 11 on photocatalytic degradation of MB dye normalized against adsorption, (B) time-dependent UV-vis absorption spectra for the adsorption and photocatalytic degradation of MB dye under optimized conditions.

Fig. 10A and S11† depict the effect of initial pH of solution (pH 3, 6 and 11) on the photoactivity of rGO-160. The corresponding % *C*_ads_, % *C*_deg_, *k* and *R*^2^ values are shown in Table 7. Throughout the experiments, the catalyst loading (60 mg), initial dye concentration (50 ppm), and light intensity (60 W m^−2^) were kept constant. Under acidic condition, there were more hydrogen ions present in the solution. The dye molecules then competed with the large amount of hydrogen ions for the unoccupied adsorption sites on the catalyst surface.^62^ It is known that MB dye can adsorb much better in basic medium as compared to acidic medium due to the cationic nature of the dye.^63^ In addition, the presence of excess hydroxyl ions also facilitated the formation of ·OH, which in turn increased the photocatalytic degradation rate.^64^ The time-dependent UV-vis absorption spectra for the removal of dye under optimized conditions are illustrated in Fig. 10B.

**Table 7:** Effect of pH on adsorption percentage, photocatalytic degradation efficiency, and photocatalytic degradation rate of MB dye

pH	Adsorption percentage (%)	Photocatalytic degradation efficiency (%)	*k* (h^−1^)	*R* ^2^
3	81.85	95.14	0.541	0.9737
6	83.45	95.29	0.545	0.9756
11	87.39	98.57	0.711	0.9998

#### 3.3.5. Recyclability test

**Fig. 11:**
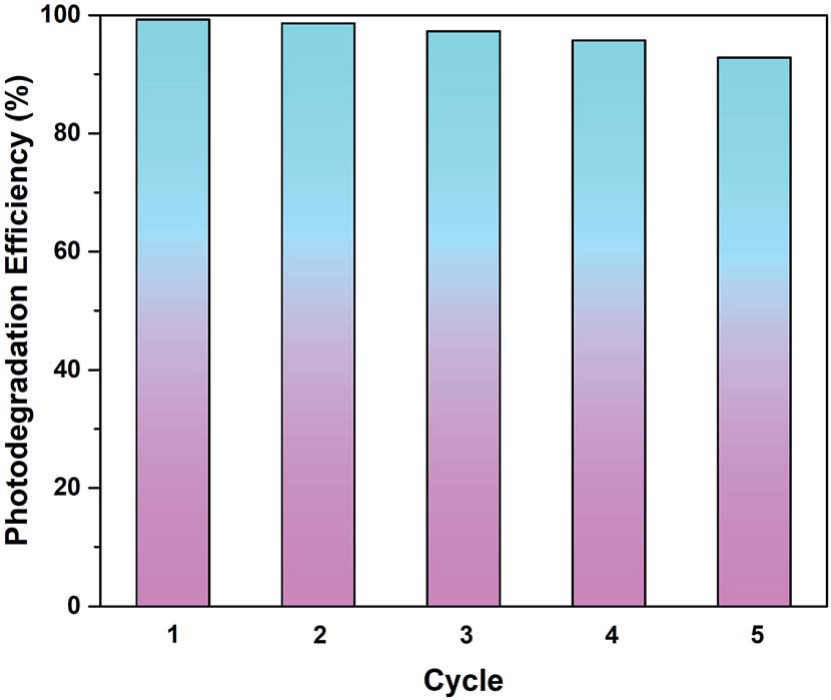
Recycling tests of rGO-160 for the photocatalytic degradation of MB dye.

From the perspective of practical applications, the recyclability of a photocatalyst is considered a crucial factor. The stability of rGO-160 was investigated for the photocatalytic dye degradation reaction as a function of number of cycles, as shown in Fig. 11. After five consecutive runs, the % *C*_deg_ was still greater than 90%, indicating that rGO-160 exhibited high stability and good reusability.

## 4. Conclusion

GO was successfully reduced in the absence of toxic reductants *via* a facile one-pot solvothermal approach. At 160 °C, the sp^2^-hybridized structure of graphene was partially restored, following the removal of surficial oxygen functionalities. The best adsorptive removal (29.26%) and photocatalytic degradation (32.68%) of MB dye were then achieved by rGO-160, which revealed that the enlargement of surface area and the narrowing of band gap were the main reasons. By optimizing the amount of catalyst to 60 mg, initial dye concentration to 50 ppm, light intensity to 60 W m^−2^, and solution pH to 11, the adsorption (87.39%) and photocatalytic activity (98.57%) of rGO-160 were further enhanced. After repeating the reaction for up to five times, rGO-160 was still able to degrade more than 90% of MB dye, indicating that it was highly stable and reusable.

## Conflicts of interest

There are no conflicts to declare.

## Supplementary Material

RA-009-C9RA05793E-s001
