# Supplementary material for: Removal of methylene blue dye by solvothermally reduced graphene oxide: a metal-free adsorption and photodegradation method
Source: RSC Adv. 2019 Nov 19;9(64):37686–95. doi: 10.1039/c9ra05793e (PMC9075724; doi:10.1039/c9ra05793e)
Supplement: RA-009-C9RA05793E-s001 [file RA-009-C9RA05793E-s001.pdf]

Electronic Supplementary Material (ESI) for RSC Advances.
This journal is © The Royal Society of Chemistry 2019

Table S1. Adsorption and photocatalytic degradation of MB dye by rGO.

Synthesis Method	Experimental conditions	Performance	Residual [MB] (ppm)	Ref.
Chemical reduction with hydrazine	rGO: 1 mg<br>[MB]: 1.3 ppm<br>Light: 100 W Hg lamp	10% adsorbed and degraded at the same time in 90 min	1.2	1
Electrochemical exfoliation from graphite electrode	rGO: 60 mg<br>[MB]: 350 ppm<br>Light: 15 W UV lamp	40% degraded in 90 min after adsorption in 4 hr	N/A	2
Chemical reduction with hydrazine	rGO: 50 mg<br>[MB]: 15000 ppm<br>Light: 100 W UV lamp	7% degraded in 100 min after adsorption of 12% in 20 min	12276	3
Chemical reduction with hydrazine	rGO: 20 mg<br>[MB]: 3.2 ppm<br>Light: Sunlight	8% degraded in 180 min after adsorption of 50% in 60 min	1.5	4
Microwave irradiation	rGO: 10 mg<br>[MB]: 10 ppm<br>Light: 250 W Hg lamp	19% degraded in 150 min after adsorption of 1% in 30 min	8	5
Photochemical reduction	rGO: 10 mg<br>[MB]: 10 ppm<br>Light: UV lamp	35% adsorbed and degraded at the same time in 120 min	6.5	6
Photochemical reduction	rGO: 10 mg<br>[MB]: 10 ppm<br>Light: 300 W Xe lamp	30% adsorbed and degraded at the same time in 120 min	7	6
Green reduction without toxic reductant	rGO: 60 mg<br>[MB]: 50 ppm<br>Light: 95 W UV lamp (x 2)	99% degraded in 6 hr after adsorption of 87% in 4 hr	0.1	This work

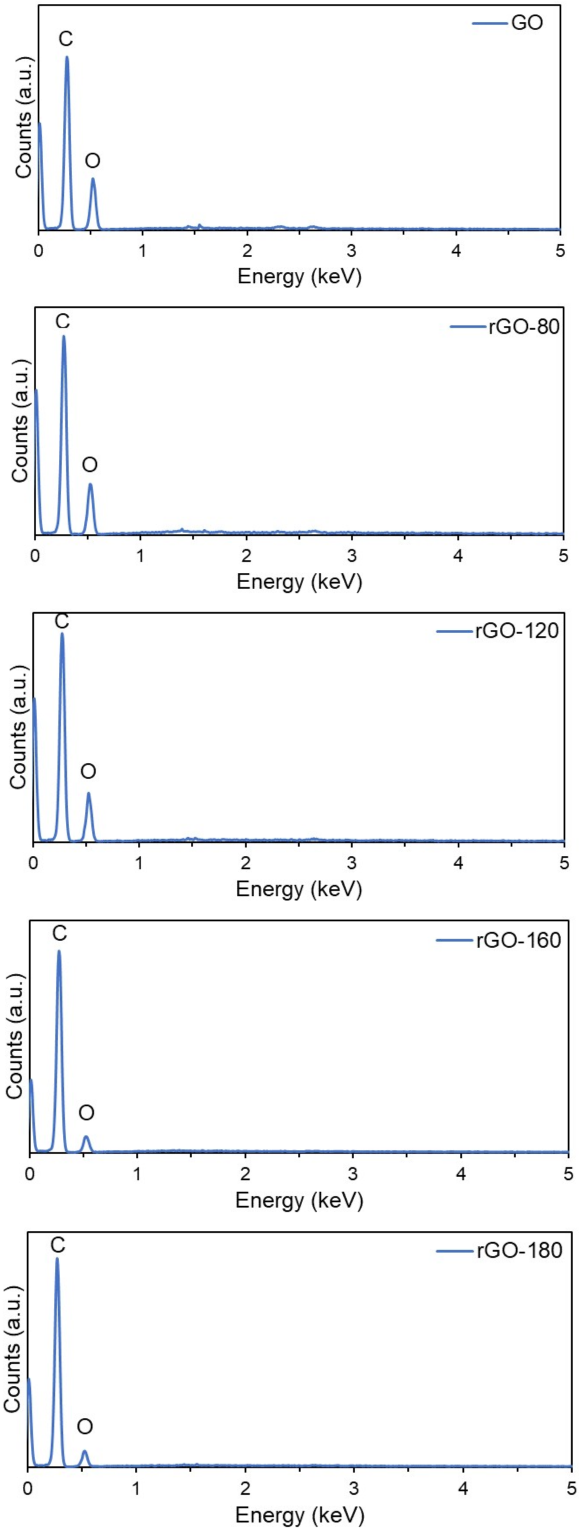

Fig. S1 EDX elemental analysis of GO, rGO-80, rGO-120, rGO-160 and rGO-180.

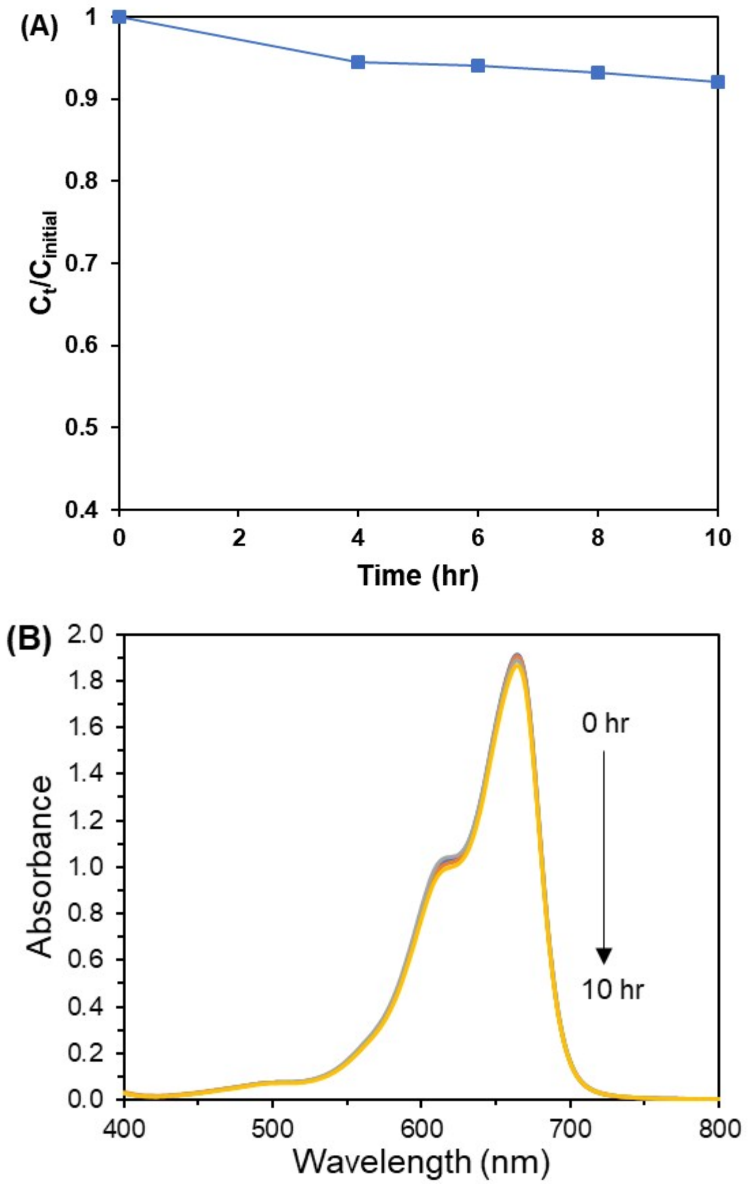

Fig. S2 (A) Removal of MB dye in the absence of photocatalyst **(B)** Time-dependent UV-Vis absorption spectra of MB dye in the absence of photocatalyst ([MB] = 50 ppm; light intensity = 60 W·m^{-2}; pH = 6).

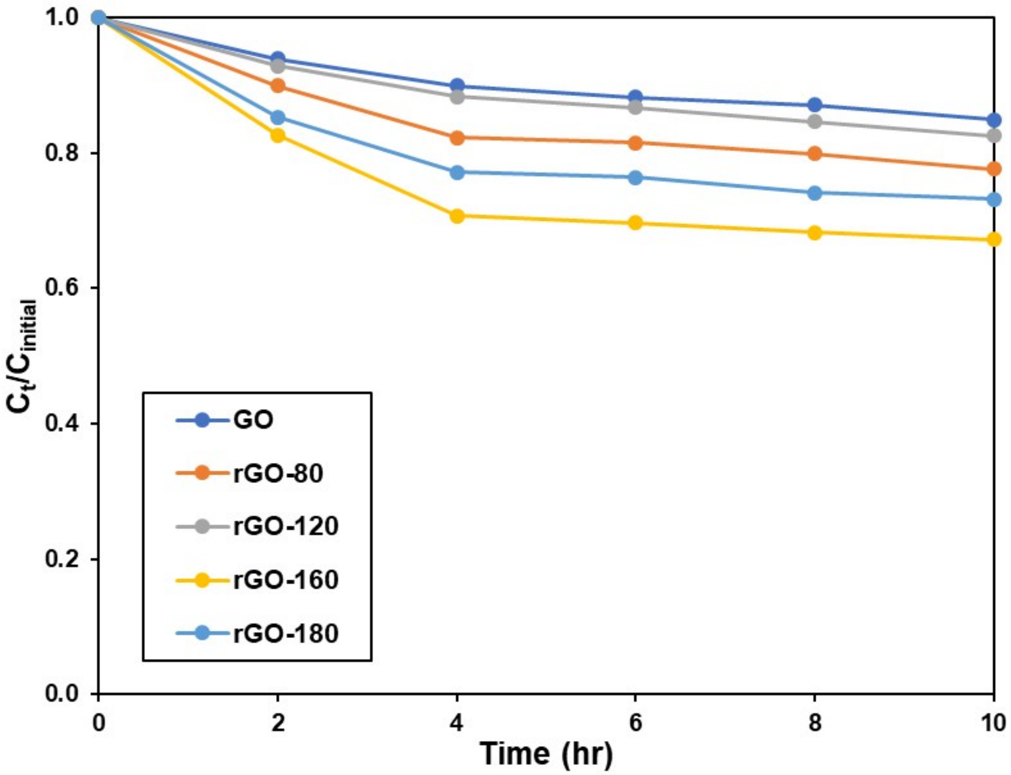

Fig. S3 Dark adsorption of MB dye by GO, rGO-80, rGO-120, rGO-160 and rGO-180 (catalyst loading = 20 mg; [MB] = 50 ppm; light intensity = 60 W·m^{-2}; pH = 6).

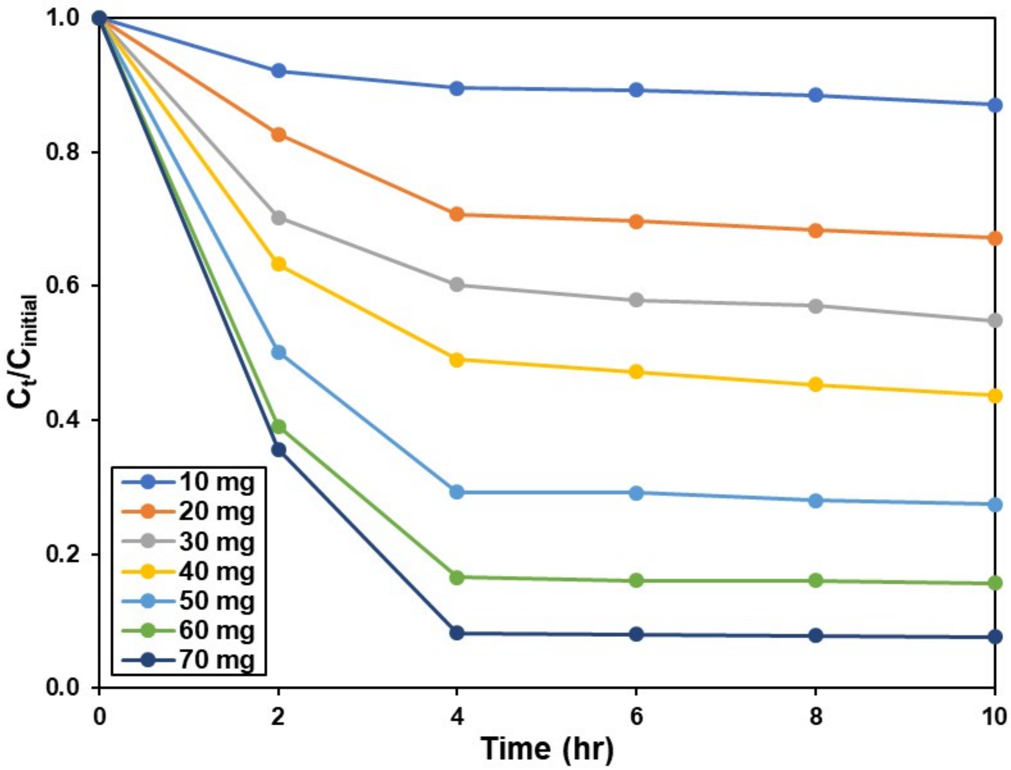

Fig. S4 Dark adsorption of MB dye by 10 mg, 20 mg, 30 mg, 40 mg, 50 mg, 60 mg and 70 mg of rGO-160 ([MB] = 50 ppm; light intensity = 60 W·m^{-2}; pH = 6).

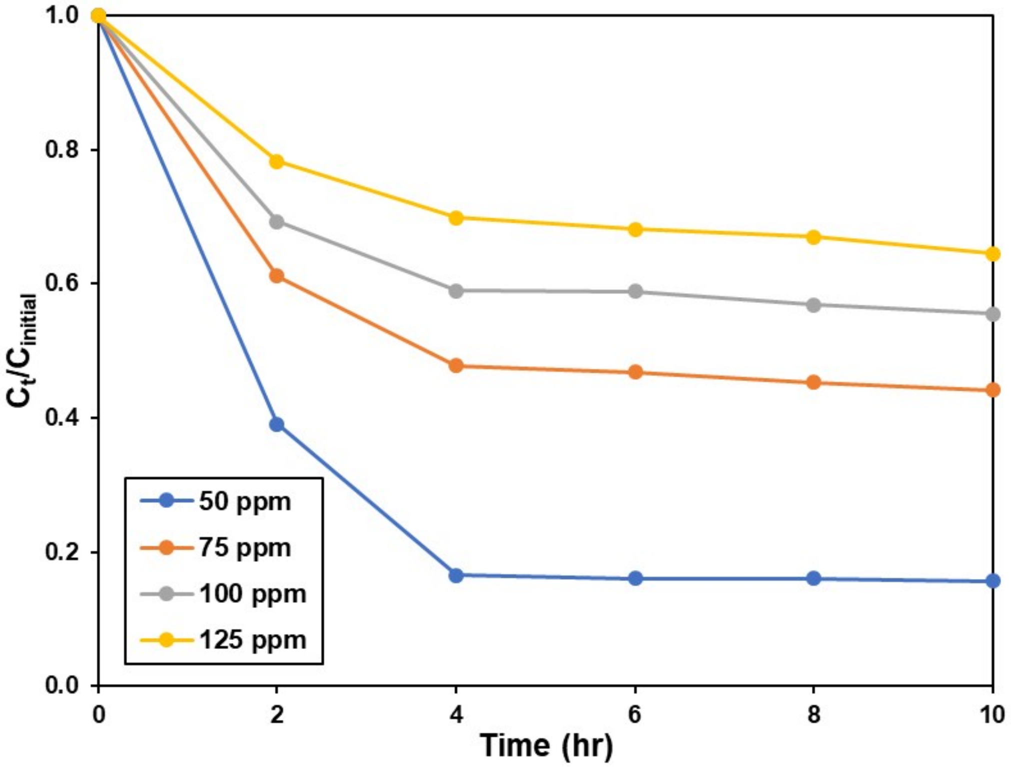

Fig. S5 Dark adsorption of MB dye by rGO-160 in 50 ppm, 75 ppm, 100 ppm and 125 ppm of MB solution (catalyst loading = 60 mg; light intensity = 60 W·m^{-2}; pH = 6).

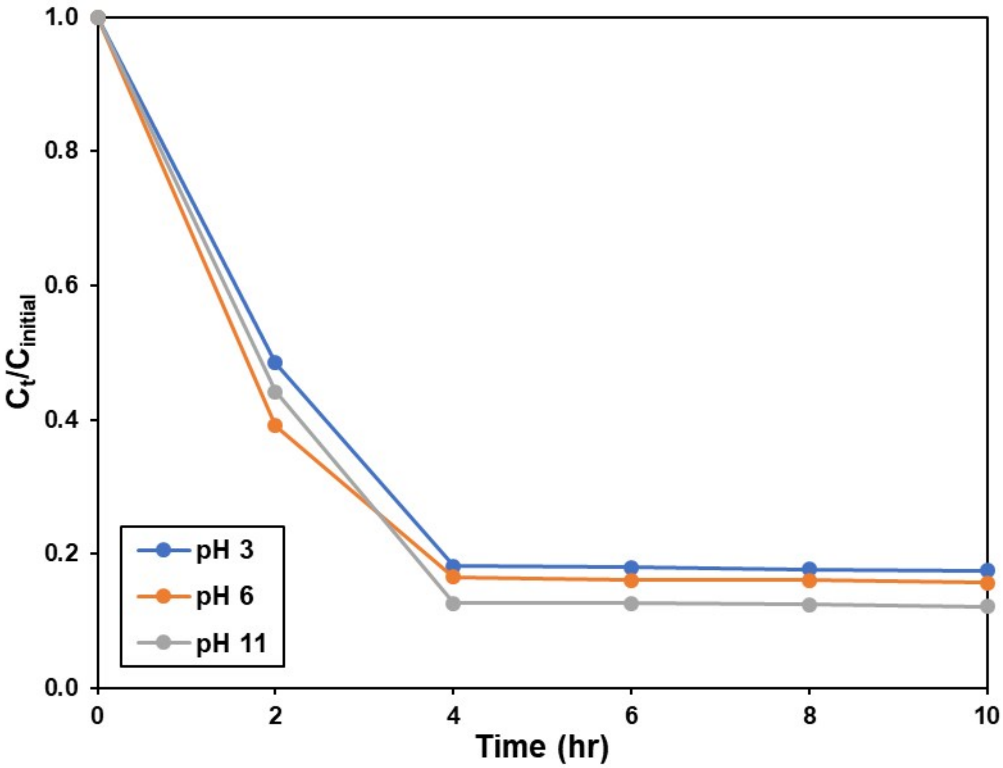

Fig. S6 Dark adsorption of MB dye by rGO-160 at pH 3, 6 and 11 of MB solution (catalyst loading = 60 mg; [MB] = 50 ppm; light intensity = 60 $W \cdot m^{-2}$).

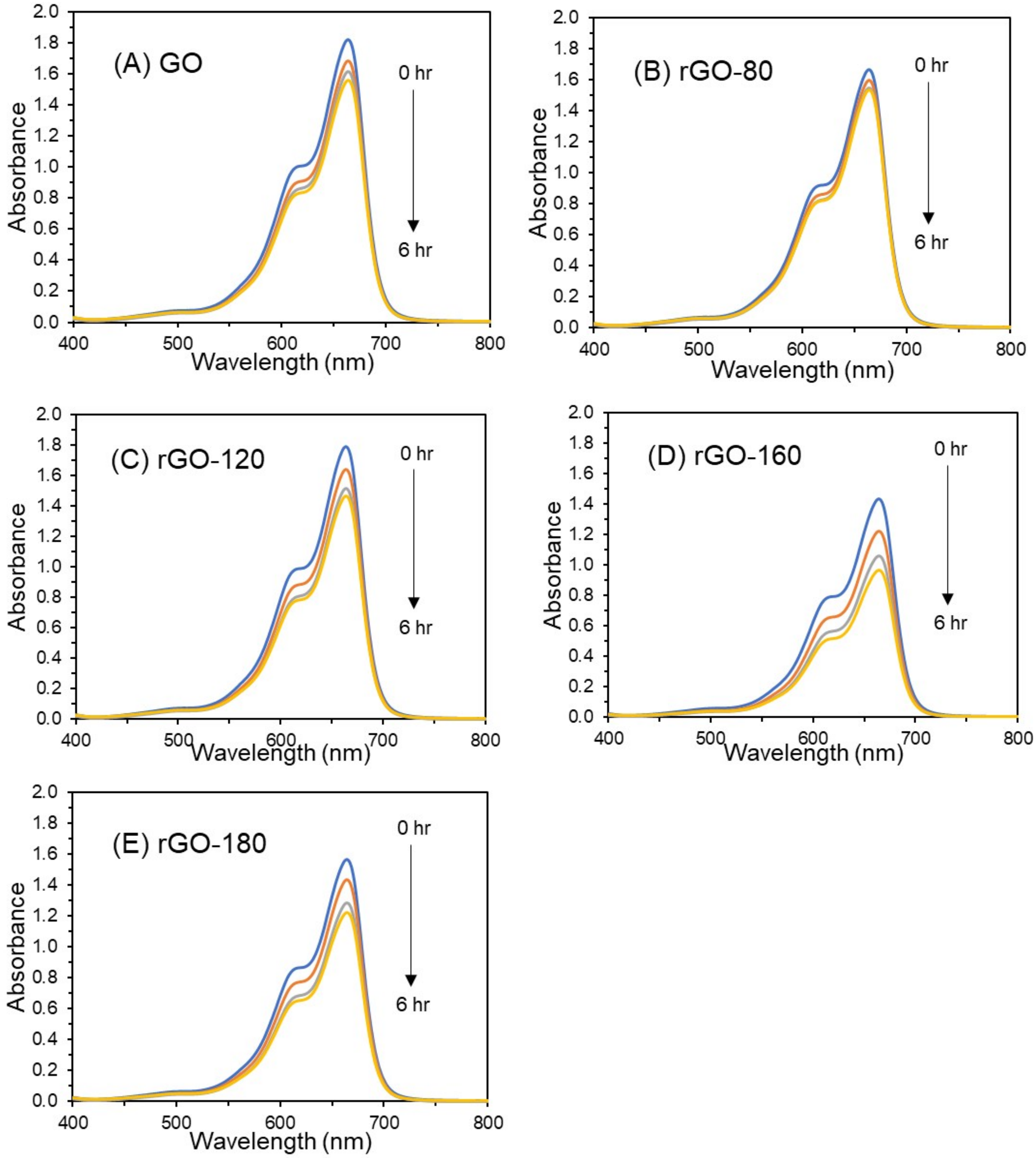

Fig. S7 Time-dependent UV-Vis absorption spectra for the photocatalytic degradation of MB dye from time, t= 0 to t=6, by different photocatalysts, where (A) GO, (B) rGO-80, (C) rGO-120, (D) rGO-160, and (E) rGO-180. (catalyst loading = 20 mg; [MB] = 50 ppm; light intensity = 60 $W \cdot m^{-2}$; pH = 6).

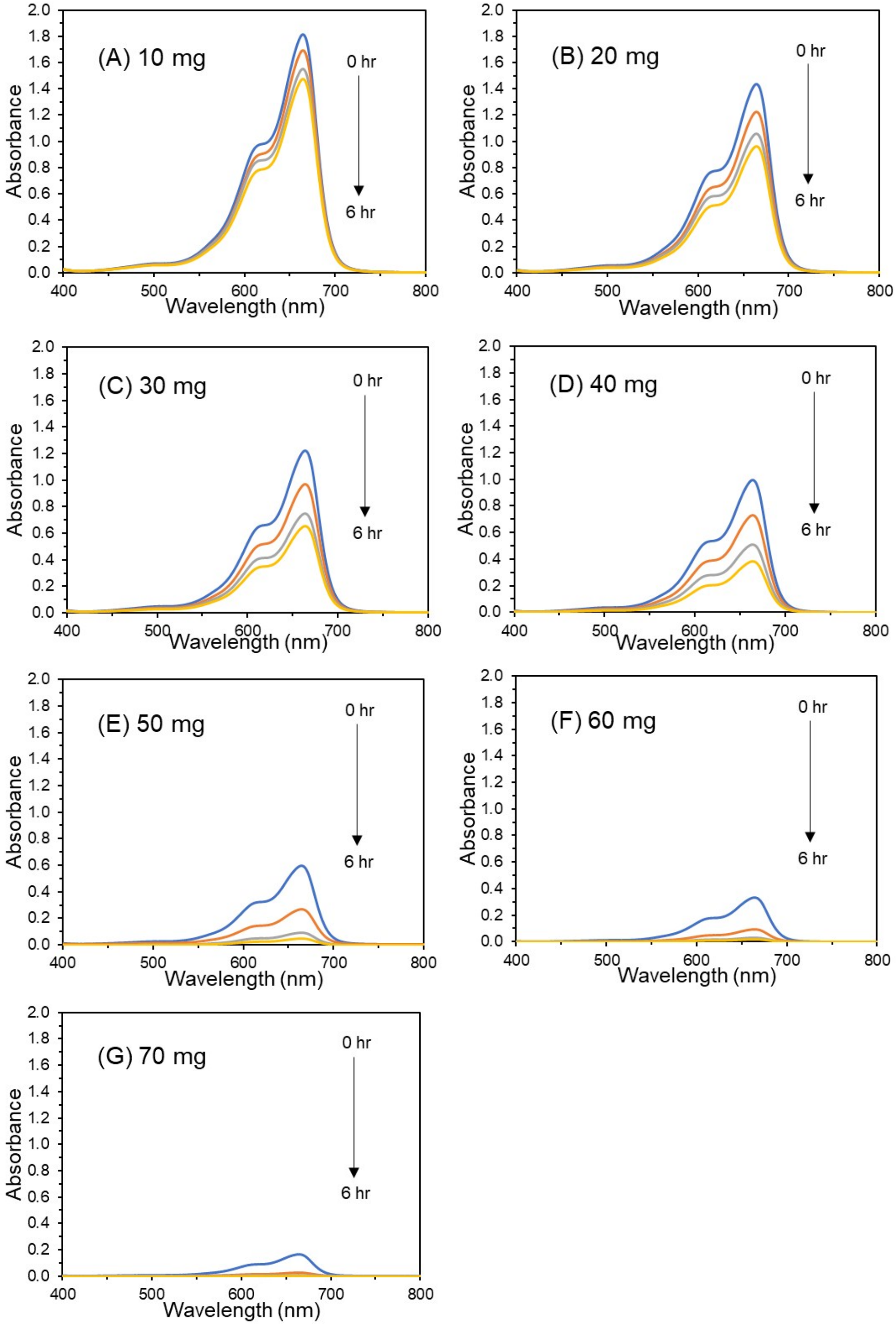

Fig. S8 Time-dependent UV-Vis absorption spectra for the photocatalytic degradation of MB dye from time, t= 0 to t=6, by rGO-160 with different catalyst loading, where (A) 10 mg, (B) 20 mg, (C) 30 mg, (D) 40 mg, (E) 50 mg, (F) 60 mg, and (G) 70 mg. ([MB] = 50 ppm; light intensity = 60 W·m^{-2}; pH = 6).

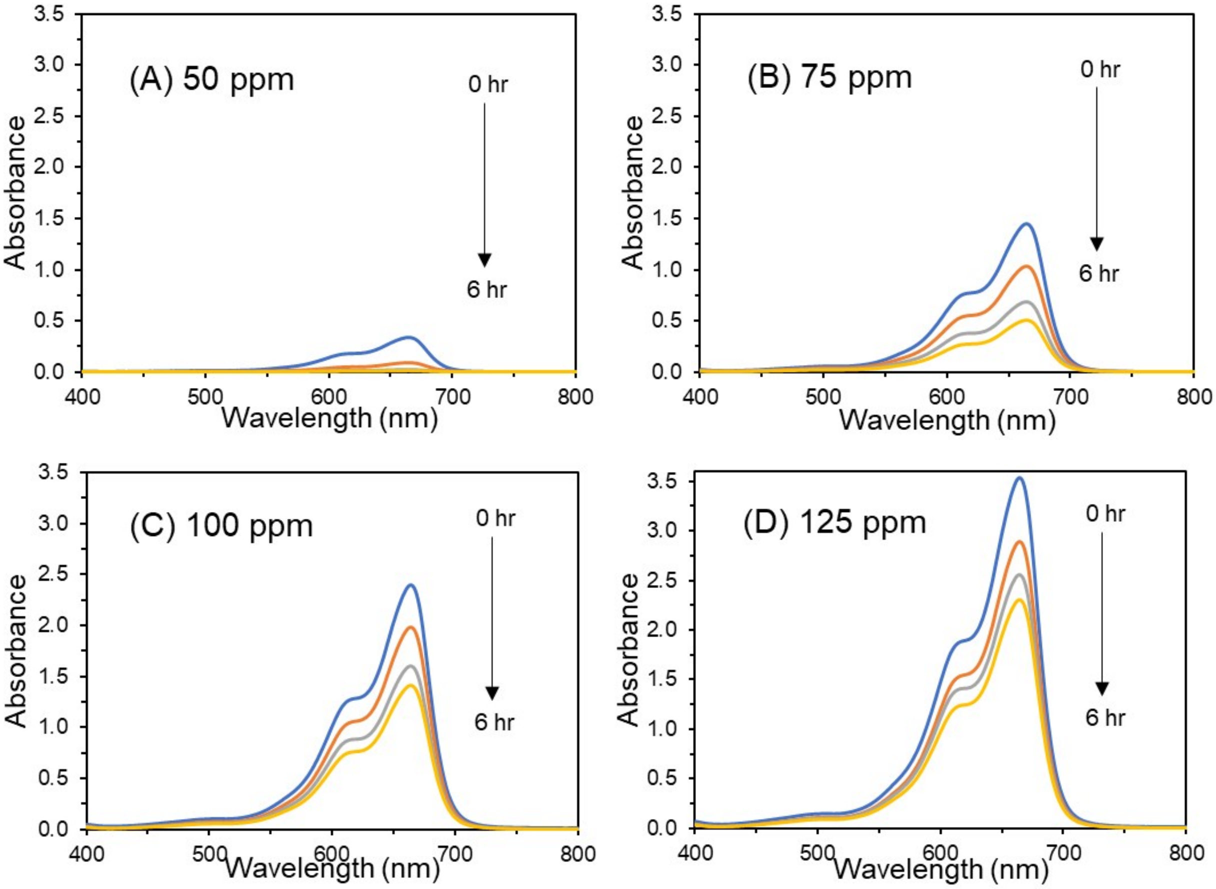

Fig. S9 Time-dependent UV-Vis absorption spectra for the photocatalytic degradation of MB dye from time, t= 0 to t=6, by rGO-160 with different initial MB concentration, where (A) 50 ppm, (B) 75 ppm, (C) 100 ppm, and (D) 125 ppm. (catalyst loading = 60 mg; light intensity = 60 W·m^{-2}; pH = 6).

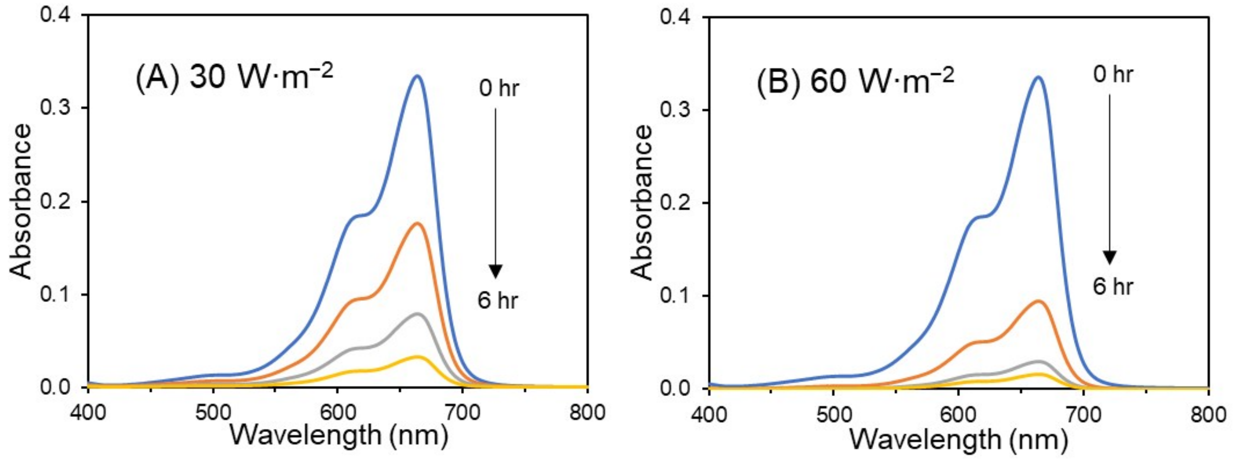

Fig. S10 Time-dependent UV-Vis absorption spectra for the photocatalytic degradation of MB dye from time, t= 0 to t=6, by rGO-160 with different light intensity, where (A) 30 W·m^{-2} and (B) 60 W·m^{-2} (catalyst loading = 60 mg; [MB] = 50 ppm; pH = 6).

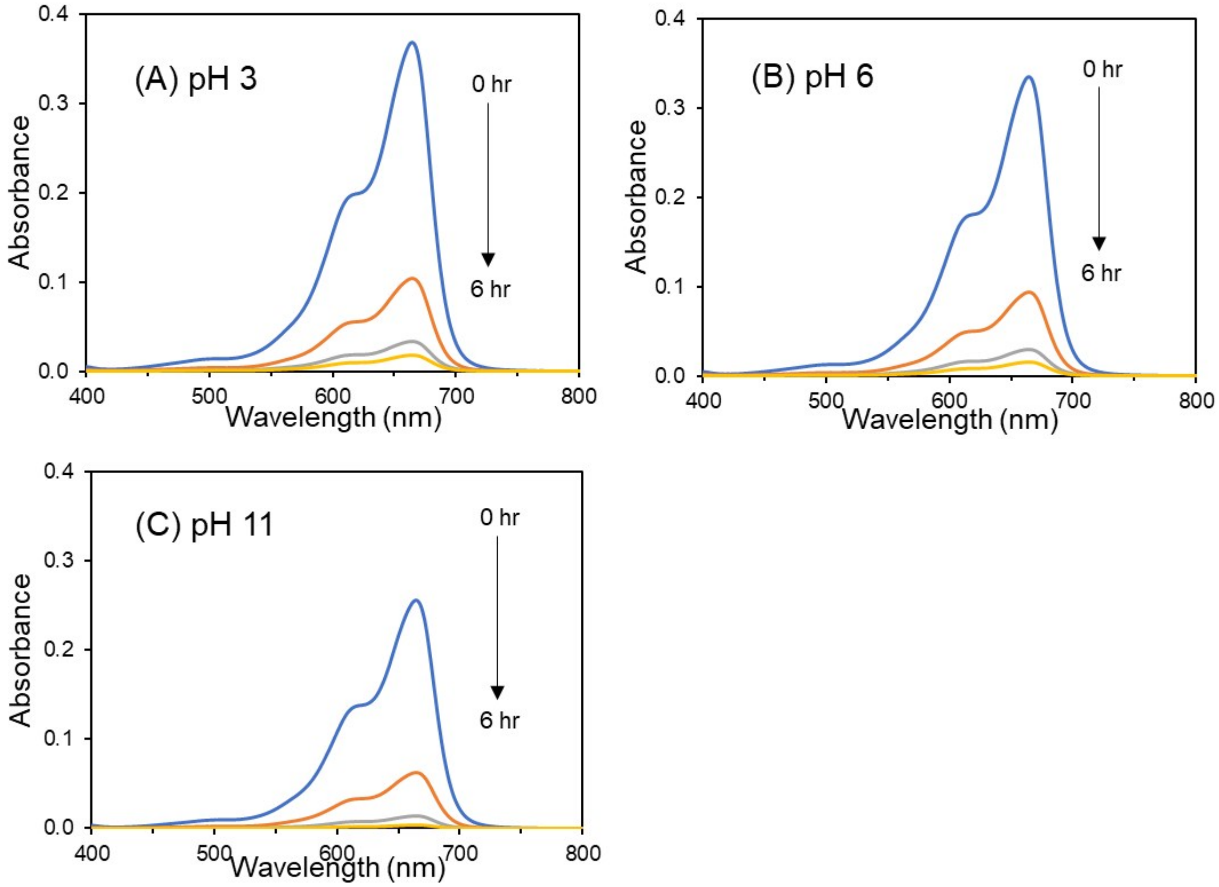

Fig. S11 Time-dependent UV-Vis absorption spectra for the photocatalytic degradation of MB dye from time, t= 0 to t=6, by rGO-160 at different pH, where (A) pH 3, (B) pH 6, and (C) pH 11 (catalyst loading = 60 mg; [MB] = 50 ppm; light intensity = 60 W·m^{-2}).

References

1. S. Chandra, P. Das, S. Bag, R. Bhar and P. Pramanik, *Mater. Sci. Eng. B*, 2012, **177**, 855-861.
2. Y. Zhao, X. Wei, Y. Wang and F. Luo, *Chem. Phys. Lett.*, 2014, **607**, 34-38.
3. S. Xu, L. Fu, T. S. H. Pham, A. Yu, F. Han and L. Chen, *Ceram. Int.*, 2015, **41**, 4007-4013.
4. J. Kaur, K. Anand, R. Thangaraj and R. Singh, *Indian J. Phys.*, 2016, **90**, 1183-1194.
5. M. J. S. Mohamed and D. K. Bhat, *AIMS Mater. Sci.*, 2017, **4**, 158-171.
6. B. Xue and Y. Zou, *J. Colloid Interface Sci.*, 2018, **529**, 306-313.
